# Comparison of Accuracy of NUTRIC and Modified NUTRIC Scores in Predicting 28-Day Mortality in Patients with Sepsis: A Single Center Retrospective Study

**DOI:** 10.3390/nu10070911

**Published:** 2018-07-17

**Authors:** Dae Hyun Jeong, Sang-Bum Hong, Chae-Man Lim, Younsuck Koh, Jarim Seo, Younkyoung Kim, Ji-Yeon Min, Jin Won Huh

**Affiliations:** 1 Department of Internal Medicine, University of Ulsan College of Medicine, Asan Medical Center, Seoul 05505, Korea; eoguseogus33@hanmail.net; 2 Division of Pulmonary & Critical Care Medicine, University of Ulsan College of Medicine, Asan Medical Center, Seoul 05505, Korea; sbhong@amc.seoul.kr (S.-B.H.); cmlim@amc.seoul.kr (C.-M.L.); yskoh@amc.seoul.kr (Y.K.); 3 Department of Pharmacy, University of Ulsan College of Medicine, Asan Medical Center, Seoul 05505, Korea; jarim.seo@amc.seoul.kr; 4 Food and Nutrition Service Team, University of Ulsan College of Medicine, Asan Medical Center, Seoul 05505, Korea; kachiya@amc.seoul.kr (Y.K.); sophiajym@hanmail.net (J.-Y.M.)

**Keywords:** modified NUTRIC Score, NUTRIC Score, nutrition risk, sepsis, intensive care unit

## Abstract

The NUTRIC (Nutrition Risk in the Critically Ill) and modified NUTRIC scores are nutrition risk assessment tools specifically for intensive care unit (ICU) patients. A modified NUTRIC score is composed of all variables except for IL-6 level in the NUTRIC score. Their use in qualifying critically ill patients at nutritional risk has been extensively evaluated, although not in studies of patients with sepsis, when interleukin 6 levels, which are not included in the modified NUTRIC score, may be elevated. The present study was a retrospective comparison of the accuracy of the NUTRIC and modified NUTRIC scores in predicting 28-day mortality of 482 adult patients with sepsis who were admitted to the medical ICU of a tertiary referral hospital in South Korea between January 2011 and June 2017 and who had ICU stays longer than 24 h. The NUTRIC and modified NUTRIC scores were calculated using data from the patients’ electronic medical records relating to the first 24 h of admission to the ICU. The area under the curve of the NUTRIC Score for predicting 28-day mortality was 0.762 (95% confidence interval (CI): 0.718–0.806) and of the modified NUTRIC Score 0.757 (95% CI: 0.713–0.801). There was no significant difference between the two scores (*p* = 0.45). The modified NUTRIC score was a good nutritional risk assessment tool for critically ill septic patients.

## 1. Introduction

Malnourished patients often have negative clinical outcomes including increased morbidity and mortality [1,2,3,4,5]. It is important to assess nutritional status and provide adequate nutritional support for critically ill patients [6]. Sepsis and septic shock are major causes of mortality among critically ill patients in the intensive care unit (ICU) [7]. Gastrointestinal dysfunction, enhanced energy expenditure, and hypermetabolism result in greater risk of malnutrition in critically ill septic patients [8]. However, there are few nutritional risk assessment protocols for ICU patients with sepsis.

Despite many studies in recent decades on the importance of adequate nutritional support for critically ill patients, the prevalence of hospital malnutrition has hardly changed during the same period. Adequate or aggressive nutritional support reduces hospital malnutrition and improves patient outcomes [9,10]. Although many nutritional risk tools, such as the Nutritional Risk Screening 2002 (NRS 2002), Subjective Global Assessment (SGA), and Short Nutritional Assessment Questionnaire (SNAQ), have been developed for outpatients and inpatients, they are unsuitable for patients in the ICU [11,12,13]. Recently, Helyland et al. have developed the Nutrition Risk in the Critically Ill (NUTRIC) Score; this is the first nutritional risk assessment tool for ICU patients and consists of age, Acute Physiology and Chronic Health Evaluation (APACHE) II score, Sequential Organ Failure Assessment (SOFA) score, number of comorbidities, days from hospital admission to ICU admission, and serum interleukin 6 (IL-6) level [14]. The NUTRIC Score helps identify critically ill patients who may receive greater benefit from aggressive nutritional therapy. In addition, Özbilgin et al. reported that the NUTRIC Score is an important indicator of morbidity and mortality in postoperative surgical patients [15]. Although the NUTRIC Score is based on variables including acute inflammation and severity of underlying illness, measurement of IL-6 levels is not performed routinely in the critical care setting. Rahman et al. have demonstrated the validity of a modified NUTRIC Score including all variables except for IL-6 level. There is a strong positive association between nutritional adequacy and 28-day mortality in patients with a high NUTRIC score, but this association diminishes with decreasing NUTRIC score [16]. Several studies have confirmed that the modified NUTRIC Score is associated with clinical outcomes [17,18]. Mukhopadhyay et al. showed that increased nutritional adequacy may reduce the 28-day mortality in patients with a high modified NUTRIC score [18]. However, it has not yet been validated in patients with sepsis, which is closely associated with IL-6 levels.

The present study was conducted to compare the accuracy of the NUTRIC Score and the modified NUTRIC Score in predicting 28-day mortality in patients with sepsis.

## 2. Materials and Methods

### 2.1. Study Participants

This study retrospectively analyzed the data of ASAN Sepsis Cohort in the medical ICU of Asan Medical Center, a tertiary referral hospital in Seoul, South Korea between January 2011 and June 2017. During this period, 518 patients who were at least 18 years old were admitted to the ICU with sepsis and had ICU stays of more than 24 h. We excluded patients who were discharged or died within 24 h, those for whom 28-day mortality could not be evaluated, and those from whom IL-6 levels from blood samples were not available; the remaining 482 patients were included in the analysis. The study was conducted in accordance with the Declaration of Helsinki and the protocol was approved by the Institutional Review Board of Asan Medical Center (IRB 2017-0833). The requirement for informed consent was waived owing to the retrospective nature of the analysis.

### 2.2. Data Collection

We reviewed patient data from the electronic medical records of Asan Medical Center and collected information on demographics, height, body mass, comorbidities, diagnosis, length of stay (LOS) in the ICU, mechanical ventilation (MV), vasopressor use, and renal replacement therapy (RRT). The NUTRIC score (0–10) and modified NUTRIC score (0–9) were calculated using data from the first 24 h after ICU admission. NUTRIC and modified NUTRIC scores ≥6 and ≥5, respectively, were considered high [14,16].

### 2.3. Statistical Analysis

We compared high and low nutritional risk using the NUTRIC and modified NUTRIC scores. Categorical variables were compared using the chi-square test and continuous variables using Student’s *t*-test or the Wilcoxon–Mann–Whitney test. The model’s discrimination for predicting 28-day mortality was assessed by the area under the receiver operating characteristic (ROC) curve for both the NUTRIC Score and modified NUTRIC Score. The ROC curves of the two scores were compared using MedCalc software (version 1.76; MedCalc Software, Ostend, Belgium). All other statistical analysis was conducted using SPSS software (version 21.0; SPSS Inc., Chicago, IL, USA). All significance tests were two-sided; a *p*-value < 0.05 was considered significant.

## 3. Results

### 3.1. Participants

Characteristics of the 482 participants are shown in Table 1 according to NUTRIC Score and modified NUTRIC Score risk groups. The median age of all participants was 66 years (interquartile range (IQR): 56–74 years), the median BMI was 23 (IQR: 20–25) kg/m^2^, and 32% of patients were female. The median APACHE II score was 21 (IQR: 16–28), the median SOFA score was 10 (IQR: 7–14), and the median number of co-morbidities was 2 (IQR: 1–3). There were 223 patients (46.3%) with neoplasms, and the median LOS in the ICU was 7 (4–14) days. MV was used in 312 (64.7%) patients, 417 patients (86.5%) received vasopressors, and 152 patients (31.5%) underwent RRT. A total 255 patients (52.9%) had high NUTRIC scores and 316 patients (65.6%) high modified NUTRIC scores.

### 3.2. Discrepancy between Scores

Among the study group, 61 patients (26.9%) had a low NUTRIC score but a high modified NUTRIC score. Of these 61 patients, the median APACHE II score was 20 (IQR: 17–23), the mean SOFA score was 9 (IQR: 7–11), and 12 patients died within 28 days of admission to the ICU.

### 3.3. 28-Day Mortality According to Score

**Figure 1:**
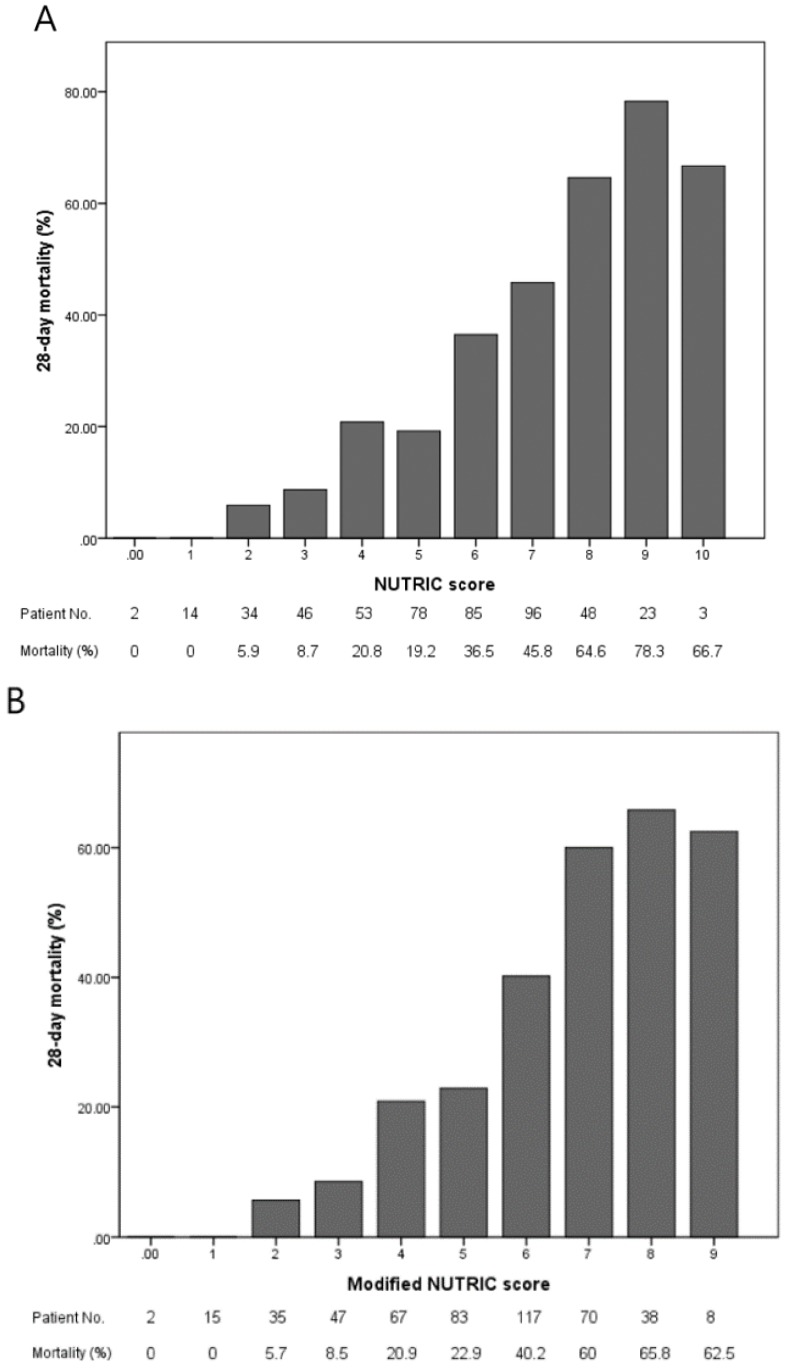
(**A**) The 28-day mortality according to NUTRIC score; (**B**) The 28-day mortality. According to modified NUTRIC score. Multivariable logistic regression analyses showed that RRT (adjusted OR: 1.91; 95% CI: 1.19–3.07; *p* = 0.007), MV use (adjusted OR: 2.96; 95% CI: 1.64–5.34; *p* < 0.001), and modified NUTRIC score (adjusted OR: 1.68; 95% CI: 1.42–1.98; *p* < 0.001) were significantly associated with higher 28-day mortality (Table S1).

Our analysis showed that 28-day mortality increased with both higher NUTRIC score and higher modified NUTRIC score (Figure 1): 28-day mortality for the maximum NUTRIC score was 66.7% and for the maximum modified NUTRIC score was 62.5% (Figure 1).

### 3.4. Area under the Curve of Scores for Predicting 28-Day Mortality

**Figure 2:**
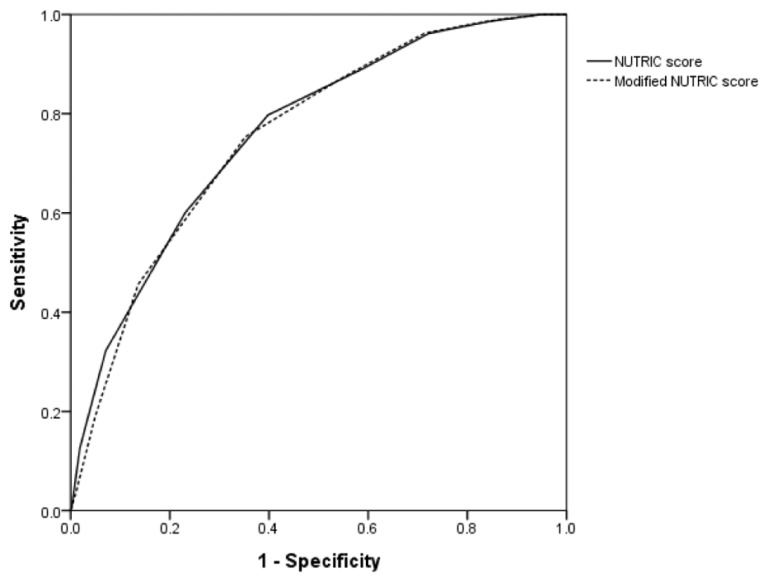
Performance of NUTRIC score and modified NUTRIC score in predicting 28-day mortality. NUTRIC score: AUC = 0.762 (95% CI, 0.718–0.806); modified NUTRIC score: AUC = 0.757 (95% CI, 0.713–0.801).

The area under the curves (AUCs) of the NUTRIC Score and modified NUTRIC Score for predicting 28-day mortality were 0.762 (95% confidence interval (CI): 0.718–0.806) and 0.757 (95% CI: 0.713–0.801), respectively (Figure 2). There was no significant difference in ROC curves between the two scores (*p* = 0.45). In the ROC curve of modified NUTRIC score, the best cutoff was at 6 (sensitivity 75% and specificity 65%), and the Youden index was 0.401 (Table 2).

**Figure 3:**
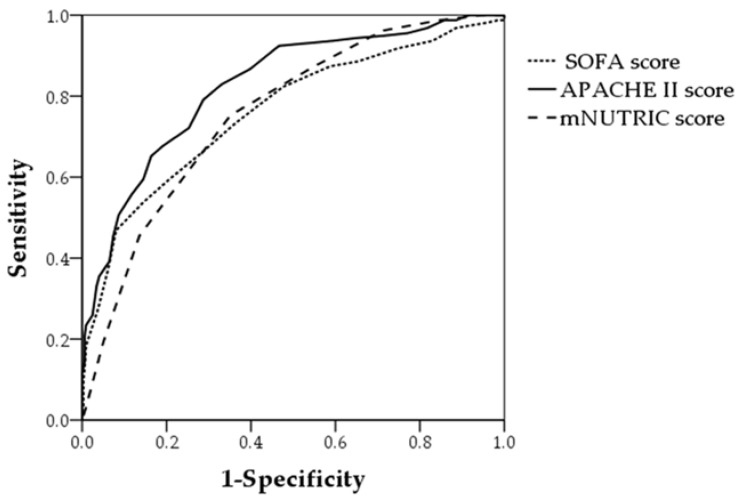
Performance of APACHE II score, SOFA score, and NUTRIC score in predicting 28-day mortality. APACHE II score: AUC = 0.826 (95% CI, 0.787–0.866); SOFA score: AUC = 0.761 (95% CI, 0.713–0.809); mNUTRIC score: AUC = 0.757 (95% CI, 0.713–0.801).

The AUCs of APACHE II score and SOFA score were 0.826 (95% CI: 0.787–0.866) and 0.761 (95% CI: 0.713–0.809), respectively (Figure 3).

## 4. Discussion

In this study, we found that the modified NUTRIC score was a good prognostic substitute for the NUTRIC score in patients with sepsis. The baseline components of the two scores are similar, except for IL-6 level. We found no significant difference between the two tools in the ability to predict 28-day mortality. Therefore, IL-6 level may not be a critical item in a nutritional risk scoring system of septic patients. A cutoff score of 6 for the modified NUTRIC score (versus a cutoff of 5) was better in predicting 28-day mortality. We classified 61 patients (26.9%) with a low NUTRIC score as having a high modified NUTRIC score; all these patients had IL-6 levels of zero, giving a score of 5 on both the NUTRIC and modified NUTRIC Scores. We postulate, therefore, that using IL-6 level as part of a nutritional assessment of septic patients may also be superfluous. Using the modified NUTRIC score enables nutritional risk to be assessed more easily, as IL-6 levels—which are not routinely measured by many institutions—are not required.

The Surviving Sepsis Campaign recommended early enteral nutrition as the preferred feeding regimen in patients with sepsis or septic shock who could be fed enterally [19]. In the NUTRIREA-2 study, early isocaloric enteral nutrition did not reduce mortality or the risk of ICU-acquired infections, but was associated with a greater risk of gastrointestinal complications compared with early isocaloric parenteral nutrition in critically ill patients in shock [20]. In the early phase of sepsis or septic shock, enteral feeding may not be feasible because of hemodynamic instability, feeding intolerance or digestive complications. In such cases, clinicians should decide whether to start parenteral nutrition early to meet nutritional goals. It is important to be able to identify ICU patients with sepsis who would benefit from adequate nutritional support, and the NUTRIC score is an important nutritional scoring system that can assess the risk of malnutrition and is, additionally, a useful prognostic marker. Özbilgin et al. reported that the modified NUTRIC score was a good predictor of morbidity and mortality in the postoperative acute care unit [15]. However, our study showed that modified NUTRIC score was as good a predictor as the NUTRIC score, of the 28-day mortality in the septic patients.

In addition, the NUTRIC score is an important nutritional risk assessment tool to guide the nutrition intervention in critically ill patients. Several studies have shown that the beneficial effects of adequate nutritional support are more evident in high-risk patients [21,22]. Heyland et al. showed that the 28-day mortality increases with the NUTRIC score, and demonstrated that patients with high NUTRIC score benefit from adequate provision of nutrition requirements, but patients with low NUTRIC score did not [14]. However, post-hoc analysis of the PermiT Trial showed that permissive underfeeding was associated with similar outcomes as standard feeding in patients with high and low score using a modified NUTRIC score [23]. These results may be due to unrestricted protein supply. Nonetheless, many studies using modified NUTRIC score showed that adequate nutrition, especially at high nutritional risk, improves the mortality [16,18,24]. The modified NUTRIC score may be helpful in guiding clinicians in providing adequate nutritional support for septic patients. Adequate nutrition reduces hospital malnutrition and improves outcomes such as morbidity and mortality.

The NUTRIC score is scored from 0 to 10 and the modified NUTRIC score from 0 to 9. When NUTRIC and modified NUTRIC scores were compared, 28-day mortality increased similarly as scores on both assessments increased. The 28-day mortality rate of patients with the maximum NUTRIC score was 66.7% and with the maximum modified NUTRIC score 62.5%. No patients with a score of 0 or 1 on either scale died. Our results are similar to those of other validation studies [14,16]. The AUC of the original development sample for predicting 28-day mortality was 0.783, which was similar to that of our study [14]. Upon comparing APACHE II score, SOFA score, and mNUTRIC score, APACHE II score was found to be a better predictor of 28-day mortality. The mNUTRIC score did not have an advantage over APACHE II score in the prediction of prognosis. However, the mNUTRIC score may be not only a predictor of prognosis but also a nutritional risk assessment tool. Unlike NUTRIC score, the mNUTRIC score can be used easily with the exception of IL-6. We found that the best cutoff of the ROC curve for modified NUTRIC score was at 6 (sensitivity 75% and specificity 64%) and the Youden index was 0.391. However, in another study, the best cutoff was at 5 (sensitivity 72% and specificity 63%, respectively) and the Youden index was 0.34 [18]. The simple method of subtracting 1 from the NUTRIC score to find the modified NUTRIC score may be inaccurate because the IL-6 level is excluded from the modified NUTRIC Score. Further investigation is needed to find the best cutoff score for the high-risk group in the modified NUTRIC Score.

This study has several strengths. To our knowledge, our study is the first to compare the NUTRIC score with the modified NUTRIC score in patients with sepsis. Second, our study is the first to suggest the best cutoff for the ROC curve of the modified NUTRIC score; contrary to previous studies, we found this was 6 in the present study.

Nevertheless, our study has several limitations. First, our study had a retrospective design and was conducted at a single center; multicenter prospective studies are needed to confirm for our findings. Second, our study was conducted among an exclusively Asian population; therefore, the results of our study may not be applicable to patients of other ethnic origins. Third, this study was conducted in a medical ICU and did not include surgical ICU patients or those from burns units; therefore, our results may not apply to all septic patients. 

## 5. Conclusions

In this study, we found no significant difference between the NUTRIC and modified NUTRIC scores in the ability to predict 28-day mortality. The modified NUTRIC score was a good nutritional risk assessment tool for critically ill septic patients. The modified NUTRIC score is a good substitute for the NUTRIC score in patients with sepsis. Further investigation is needed to evaluate the cutoff for the modified NUTRIC score in predicting 28-day mortality.

## Figures and Tables

**Table 1:** Patient characteristics according to NUTRIC score and modified NUTRIC score.

Variable	NUTRIC Score (*n* = 482)			Modified NUTRIC Score (*n* = 482)		
	Low Score (*n* = 227)	High Score (*n* = 255)	*p*-Value	Low Score (*n* = 166)	High Score (*n* = 316)	*p*-Value
Age, years	63 (52–72)	68 (57–75)	<0.001	62 (48–71)	68 (57–75)	<0.001
Height, cm	163 (158–170)	163 (155–170)	0.175	163 (158–170)	163 (155–170)	0.456
Weight, kg	59 (52–66)	58 (51–67)	0.945	59 (52–67)	59 (52–66)	0.842
BMI, kg/m^2^	23 (20–25)	23 (20–25)	0.456	23 (20–25)	22 (20–25)	0.827
Female, *n* (%)	68 (30)	86 (33.7)	0.376	52 (31.3)	102 (32.3)	0.831
APACHE II score	16 (13–20)	27 (22–32)	<0.001	15 (12–18)	25 (21–31)	<0.001
SOFA score	7 (5–10)	13 (11–16)	<0.001	6 (5–9)	12 (10–15)	<0.001
Days from hospital to ICU	0 (0–0)	0 (0–9)	<0.001	0 (0–0)	0 (0–8)	<0.001
Co-morbidities	1 (1–2)	2 (1–3)	<0.001	1 (1–2)	2 (1–3)	<0.001
IL-6, pg/mL	71 (21–169)	366 (54–1910)	<0.001			
LOS in ICU, days	5 (3–9)	9 (4–17)	<0.001	5 (3–9)	8 (4–17)	<0.001
MV	102 (44.9)	210 (82.4)	<0.001	65 (20.8)	247 (78.2)	<0.001
Vasopressor use	172(75.8)	245 (96.1)	<0.001	123 (74.1)	294 (93.0)	<0.001
RRT	26 (11.5)	126 (49.4)	<0.001	17 (10.2)	135 (42.7)	<0.001
Diagnosis			0.693			0.465
Respiratory disease	103 (45.4)	118 (46.3)		69 (41.6)	152 (48.1)	
Liver/GI disease	60 (26.4)	63 (24.7)		48 (28.9)	75 (23.7)	
Cardiovascular disease	4 (1.8)	6 (2.4)		2 (1.2)	8 (2.5)	
Renal disease	22 (9.7)	18 (7.1)		17 (10.2)	23 (7.3)	
Febrile neutropenia	14 (6.2)	14 (5.5)		9 (5.4)	19 (6)	
SSTI	10 (4.4)	10 (3.9)		9 (5.4)	11 (3.5)	
Other	14 (6.2)	26 (10.2)		12 (7.2)	28 (8.9)	

BMI, body mass index; APACHE, Acute Physiology and Chronic Health Evaluation; SOFA, Sequential Organ Failure Assessment; TIA, transient ischemic attack; CRP, C-reactive protein; LOS, length of stay; ICU, intensive care unit; MV, mechanical ventilation; RRT, renal replacement therapy; GI, gastrointestinal; SSTI, skin and soft tissue infection. Data are presented as number (%) or median (IQR).

**Table 2:** Change in sensitivity and specificity according to cutoff.

	NUTRIC Score	mNUTRIC Score (Cutoff = 5)	*p*-Value	mNUTRIC Score (Cutoff = 6)	*p*-Value
Sensitivity	0.797	0.873	<0.001	0.753	0.016
Specificity	0.602	0.451	<0.001	0.648	<0.001
Youden index	0.399	0.324		0.401	

mNUTRIC score, modified NUTRIC score.

## Supplementary Materials

The following are available online at http://www.mdpi.com/2072-6643/10/7/911/s1, Table S1: Univariable and multivariable logistic regression analysis for 28-day mortality.
