# Supplementary material for: Comparison of Accuracy of NUTRIC and Modified NUTRIC Scores in Predicting 28-Day Mortality in Patients with Sepsis: A Single Center Retrospective Study
Source: Nutrients. 2018 Jul 17;10(7):911. doi: 10.3390/nu10070911 (PMC6073879; doi:10.3390/nu10070911)
Supplement: Supplementary file 1 [file nutrients-10-00911-s001.pdf]

Supplementary Table 1. Univariable and multivariable logistic regression analyses for 28-day mortality

	Univariable analysis		Multivariable analysis	
**Variables**	**Crude OR (95% CI)**	**P-value**	**Adjusted OR (95% CI)**	**P-value**
Age	0.997 (0.983-1.010)	0.628	0.969 (0.952-0.987)	0.001
Sex	1.321 (0.883-1.975)	0.175	..	..
Vasopressor use	5.640 (2.379-13.372)	<0.001	..	..
RRT	4.466 (2.957-6.746)	<0.001	1.909 (1.189-3.065)	0.007
MV use	6.873 (4.017-11.760)	<0.001	2.958 (1.639–5.341)	<0.001
Diagnosis	1.183 (0.597-2.341)	0.630	..	..
Modified NUTRIC score	1.814 (1.575-2.090)	<0.001	1.676 (1.416-1.983)	<0.001

MV, mechanical ventilation; RRT, renal replacement therapy
